# Skeletal Muscle Metabolism in Duchenne and Becker Muscular Dystrophy—Implications for Therapies

**DOI:** 10.3390/nu10060796

**Published:** 2018-06-20

**Authors:** Ahlke Heydemann

**Affiliations:** 1 Department of Physiology and Biophysics, University of Illinois at Chicago, Chicago, IL 60612, USA; ahlkeh@uic.edu; Tel.: +1-312-355-0259; Fax: +1-312-996-1414; 2 Center for Cardiovascular Research, The University of Illinois at Chicago, Chicago, IL 60612, USA

**Keywords:** muscular dystrophy, skeletal muscle metabolism, mitochondria, sarcolemma

## Abstract

The interactions between nutrition and metabolism and skeletal muscle have long been known. Muscle is the major metabolic organ—it consumes more calories than other organs—and therefore, there is a clear need to discuss these interactions and provide some direction for future research areas regarding muscle pathologies. In addition, new experiments and manuscripts continually reveal additional highly intricate, reciprocal interactions between metabolism and muscle. These reciprocal interactions include exercise, age, sex, diet, and pathologies including atrophy, hypoxia, obesity, diabetes, and muscle myopathies. Central to this review are the metabolic changes that occur in the skeletal muscle cells of muscular dystrophy patients and mouse models. Many of these metabolic changes are pathogenic (inappropriate body mass changes, mitochondrial dysfunction, reduced adenosine triphosphate (ATP) levels, and increased Ca^2+^) and others are compensatory (increased phosphorylated AMP activated protein kinase (pAMPK), increased slow fiber numbers, and increased utrophin). Therefore, reversing or enhancing these changes with therapies will aid the patients. The multiple therapeutic targets to reverse or enhance the metabolic pathways will be discussed. Among the therapeutic targets are increasing pAMPK, utrophin, mitochondrial number and slow fiber characteristics, and inhibiting reactive oxygen species. Because new data reveals many additional intricate levels of interactions, new questions are rapidly arising. How does muscular dystrophy alter metabolism, and are the changes compensatory or pathogenic? How does metabolism affect muscular dystrophy? Of course, the most profound question is whether clinicians can therapeutically target nutrition and metabolism for muscular dystrophy patient benefit? Obtaining the answers to these questions will greatly aid patients with muscular dystrophy.

## 1. Introduction

Metabolism encompasses a broad spectrum of biological events. Metabolism occurs at all levels, from macro diet and nutritional metabolic characteristics, to molecular-level mitochondrial health; downstream detrimental side-effects from unregulated metabolism, such as excessive reactive oxygen species, and downstream beneficial side-effects (such as potential fiber-type switching); and a higher rate of metabolism with chronic exercise. This review will concentrate upon the nutritional and cellular metabolism of skeletal muscles, as these pathways will be among those that are clinically targeted to treat muscular dystrophy (MD).

There are many excellent reviews regarding the many genes, which when mutated cause MD [1]. Therefore, only a brief introduction to the dystrophin mutation will be provided. Mutations in dystrophin cause Duchenne (DMD) and the milder Becker MD in humans. DMD is the most common form of MD, and is very severe in patients. The dystrophin mutation is naturally modeled in *mdx* mice [2]. Because the *mdx* mouse presents a very mild MD [3], additional breeding and knockout mice have been generated to increase disease severity, which better models the human disease. These include breeding the *mdx* mice with the utrophin (*utr*) knockout mice, to generate double knock-out mice (DKO, [4]), and breeding the *mdx* mice [5,6] onto the highly fibrotic DBA 2/J mouse strain [7,8]. Interesting metabolic work has also been conducted on mice modeling other forms of muscular dystrophy. However, the results and mechanisms are too broad to cover here. 

**Figure 1:**
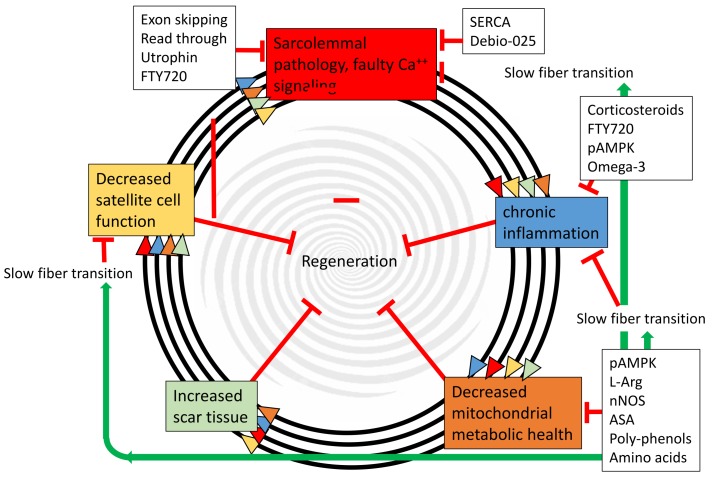
Muscular dystrophy progression is a feed-forward spiral between sarcolemmal pathology, chronic inflammation, decreased mitochondrial health, increased scar tissue, and decreased satellite cell function. The multiple arrows indicate that each of the five pathogenic steps directly accelerates all of the other steps, and all five directly inhibit regeneration. Black boxes indicate therapies directed at specific targets. Importantly, many of these therapies are nutritional/metabolic and can be used in conjunction with other therapies. Green arrows indicate positive effects, and red tees indicate inhibitive affects. pAMPK—phosphorylated AMP-activated protein kinase; l-Arg—l-Ariginine; nNOS—neuronal nitric oxide synthase; ASA—5-aminosalicylic acid; FTY720—Fingolimod; SERCA—sarco/endoplasmic reticulum Ca^2+^-ATPase; Debio-025—a cyclophilin inhibitor.

MD disease progression is largely the same in humans and in the select mouse models that produce a severe phenotype [6,9]. The disease commences with sarcolemmal membrane tears, which quickly cause sarcolemmal fragility, and increased basal Ca^++^ in the cells, which leads to inappropriate cell signaling. The membrane fragility and increased Ca^++^ cause the muscle cells to weaken. In addition, the damaged sarcolemma causes a pathogenic chronic inflammation [10]. These pathologies result in repeated rounds of degradation and regeneration. Eventually, the regeneration cannot keep pace and the cells undergo a complete necrosis and are replaced by fibroblasts, myofibroblasts, immune cells, and sometimes, adipose cells. The fibroblasts and myofibroblasts produce excessive extra cellular matrix and scar tissue, which eventually become permanent (reviewed in [11] and illustrated in Figure 1). As this fibrosis progresses in the heart or diaphragm, it becomes fatal in humans. In humans, this well-described pathogenic progression aligns with a consistent phenotypic progression [12]. MD is diagnosed at approximately 2 years old and as weakening progresses, corticosteroid therapy is initiated around 6 years old. As the disease progresses further and the skeletal muscles continue to weaken, the children lose ambulation at about 12 years old. At about 19 years, the weakened diaphragm causes the necessity for nighttime assisted ventilation. At several U.S. clinics, 5–10 year old the children begin prophylactic treatment for heart failure. Between 20 and 40 years old, the patients die from cardiac or respiratory deficiencies. The combined available treatments (corticosteroids, assisted nighttime ventilation, and heart medications) have extended some patients’ lives to 35 years old, but an effective treatment for this devastating disease still eludes the research community, clinicians, and patients. Therefore, scientists and clinicians are investigating new molecular targets; historically and again recently, metabolic signaling cascades have been on the list of possible therapeutic targets.

An open question is as follows: how much does metabolism affect MD? Because nutrition and metabolism affect all human diseases and conditions, metabolism must also significantly affect MD. Clearly metabolism is affected by nutrition and both can therefore be utilized to decrease the severe pathology of MD. And to fully understand the nutritional goals of therapy, one must consider the cellular metabolic mechanisms. Multiple investigators have hypothesized that MD is primarily a metabolic disease and the sarcolemmal damage is a downstream sequela (reviewed in [13]). In either case (whether metabolic changes are pathogenic or not), treating the metabolic pathologies that are found in *mdx* and DMD are known to provide therapeutic benefit and therefore must be pursued. In addition, because MD is a systemic disease that involves skeletal muscle myopathy, cardiac muscle myopathy, chronic inflammation, aberrant signaling (calcium, nitric oxide, and TGFβ, to name a few examples), and aberrant metabolism, the treatment options need to consider all of the pathologic mechanisms and therefore co-therapies must be researched and brought to the clinics [14,15,16]. Further important questions are as follows: Which aspects of metabolism are being overlooked? Which other metabolic mechanisms can be targeted for the treatment of MD? The answers to these issues must be expedited for patient benefit. This review attempts to summarize the current body of knowledge regarding nutrition and metabolism in MD to support future research into this dynamic and important topic.

## 2. The Interacting Nutritional and Pathological Aspects of MD

Nutrition and metabolism have a complex reciprocal relationship, specifically with muscle. Muscle utilizes the majority of the digested calories and therefore in MD, when these calories are not being utilized, excessive storage can cause pathogenic weight gain [17]. Young MD patients have increased weight compared with normal children, while older MD patients have decreased weight [17,18]. In addition, the weight gain in the young patients is exaggerated as a result of an additional loss of lean muscle mass in MD patients, indicating that the relative non-lean weight gain is substantially increased [19]. The muscle loss may arise from a number of factors. It is speculated that disuse and atrophy are the major contributors to this pathology. It can also be speculated that the overall weight gain is due to inactivity. Corticosteroids also cause weight gain [17]. The older MD patients are likely lose weight because of constipation, difficulty chewing, difficulty swallowing, and a requirement for increased calories for muscle regeneration [17]. In addition, because MD patients are living longer, additional nutritionally associated pathologies are expected to be revealed. For instance, Salera et al. discuss the consequences of nutritionally impacted pathologies, such as potential gastrointestinal issues, osteoporosis, continued weight loss, or metabolic syndrome, which will become more problematic as the patients’ lives are extended [17]. Strategies to treat these many MD macro nutrition-related ailments are also discussed in Salera et al.; among the guidelines that have been discussed are careful calorie management, awareness of difficulties swallowing, increased risks of aspirating, and careful nutritional guidance to reduce bone fragility. In addition, as patients are living longer, further side-effects of chronic corticosteroid use will likely be exposed.

## 3. Normal Muscle Cellular Metabolism

There are two main types of skeletal muscle cells with different metabolic and contraction characteristics [20,21,22]. The slow, Type 1 fibers perform a high percentage of oxidative phosphorylation, require constant oxygen, produce ATP over a long period of time, and are used for chronic work. The fast, Type 2 fibers, prefer glycolysis and not every generated pyruvate molecule progresses into the mitochondria for the citric acid cycle and oxidative phosphorylation. Type 2 fibers are used during anaerobic exertions when oxygen is scarce, they produce a lot of quick contraction power, but cannot sustain long periods of exercise. The Type 2 fibers also rely upon creatine phosphate for energy bursts. There are also intermediate fiber types (Type 2a) that have the oxidative capability similar to the Type 1 fibers and the susceptibility to fatigue that is normally seen in Type 2 fibers. Most muscle groups are a mixture of the types. However, a few muscles are predominantly slow like the soleus muscle or predominantly fast like the extensor digitorum longus muscle. Very interestingly, fiber type characteristics are plastic and can change depending upon exercise and medications, and to a lesser extent, age, gender, and nutritional differences [22,23,24]. The fact that muscle cells are plastic and can modulate metabolic and contraction characteristics is important as slow muscle fibers are spared from severe MD pathology [25], and if the fiber type can be altered therapeutically, this will provide great functional benefits to the patients.

There are three macromolecules that muscle cells utilize as sources of energy, carbohydrates, lipids, and amino acids. Carbohydrates in the form of glucose and glycogen are the main energy sources. Moderate exercises increase the carbohydrate use and cause some energy to be derived from lipid oxidation. Intense exercises reduce lipid oxidation and utilize some energy from lactate and phosphocreatine stores [26]. Exercise is a very good example of how skeletal muscle can affect systemic metabolism by requiring additional calories. The opposite is also true; a sedentary lifestyle affects systemic metabolism by requiring fewer calories and causing the storage of excess calories.

In addition to eliciting substrate changes, chronic aerobic exercise is also an example of the above mentioned plasticity of muscle cells. Chronically exercised skeletal muscles become more oxidative [24,27,28]. The truly fascinating aspect is that the fibers have the ability to transition [29], and this plasticity can be harnessed for MD treatments. An interesting aspect that requires more investigation is how the transitioning muscle cells coordinately convert their structural sarcomeric proteins and their metabolic proteins [20,30,31]. These investigations will provide additional MD therapeutic targets, and possibly biomarkers for disease progression, and to assess therapeutic benefits.

## 4. Dystrophic Muscle Cellular Metabolism

There are a number of metabolic changes observed in *mdx* mice, double knockout *mdx*/*utrophin*^−/−^ (DKO) mice, and DMD patients. An open question is whether these changes are pathogenic and harm the individual, whether they are compensatory and benefit the mouse or human, or whether the compensatory changes ultimately become pathogenic, as is often observed with cardiac decompensation. From the current data—discussed below—it appears that the metabolic changes are compensatory, perhaps to increase the energy production that is required for the constant regeneration that is required, and do not progress into decompensation.

It has been well demonstrated that slow fibers, and muscles that are composed mostly of slow fibers, are resistant to severe MD pathology (mice [32], and humans [25]). In addition, one of the major compensatory occurrences in dystrophic muscle is the transition to slow fiber type [4,33,34]. Interestingly, the transition to slow fibers is not complete and some intermediate fiber type characteristics predominate [35]. For example, the newly transitioned slow fibers upregulate hexokinase 1 and pyruvate kinase M1, usual indictors of fast Type 2 fibers, however these same fibers increase their oxygen usage and prefer the metabolic substrate pyruvate; indicating the formation of a mixed fiber type in the muscle groups that are usually fast fiber type [35]. This adaptation appears beneficial since the slow fibers are expected to maintain function better than fast fibers, and to be somewhat resistant to necrosis.

There are also many current investigations attempting to identify which slow fiber characteristics provide a portion of MD resistance. It is most likely a combination of slow fiber characteristics that provide the benefit. (1) A well-known characteristic is the natural increase of utrophin in slow fibers [36]. Utrophin is structurally similar to dystrophin and can therefore partially functionally compensate for the missing dystrophin [36,37]; (2) Slow fibers also discharge less reactive oxygen species (ROS) [38], a highly reactive molecule that is capable of damaging many cellular macromolecules when its levels are increased [39]; (3) Slow fibers are resistant to fatigue and may therefore be resistant to the extremes of contraction induced injury [34]; (4) Slow fibers also contain more activated AMP-activated protein kinase (pAMPK), which is protective of cells through a number of mechanisms [40,41,42]. Therefore, the natural plasticity of muscle and fiber type switching are very important for reduced MD pathology, and these muscle characteristics can be exploited for effective MD therapies.

In addition, many of the factors that cause a slow fiber transition likely aid dystrophic muscles through other mechanisms as well. For example, pAMPK is known to also decrease fibrosis by decreasing TGFβ signaling [43], increasing the mitochondrial amount [44], and increasing autophagy [40]. Data also indicates that there is a natural—probably compensatory—increase of pAMPK levels in *mdx* mice [45].

Interesting studies have indicated that the benefits of slow fiber upregulation are likely a combination of the characteristics that have been listed above. For example, even in the double knock-out *mdx/utr* (DKO) mice, there is still some benefit to fiber type switching, despite an inability to upregulate utrophin. The DKO mice compensate for MD pathology by upregulating slow muscle fiber characteristics at 8 weeks of age [4,46]. This includes normally fast muscle bundles (extensor digitorum longus) becoming fatigue resistant, and using more oxygen to generate force [35]. The proteins increasing the remodeling of mitochondria through fission and fusion were also found to be increased [35]. Despite the switch to slow fiber type kinetics, these mice tended to utilize increased glycolytic and decreased oxidative phosphorylation for energy [35], perhaps to compensate for the increased energy demands for regeneration. These studies therefore identified that utrophin upregulation is important, but is not the only benefit of fiber type switching.

An equally important question is which molecular pathways are being utilized to cause the fiber transition? (1) The transition to slow fibers may be orchestrated to maintain skeletal muscle ATP production. This would involve the upregulation of pAMPK and the resultant increases in mitochondria and oxidative phosphorylation; (2) The transition may also be a survival of the fittest situation; the slow fibers are not as damaged, therefore, they may predominate after a certain amount of turnover; (3) There are likely other pathways that also push fiber transition.

Recently, it was demonstrated that mitochondrial dysfunction is an early MD phenotype, and that these impaired mitochondria are susceptible to further damage after sarcolemmal injury ([47] and reviewed in [13]). An often-documented metabolic difference is the decreased number of mitochondria in *mdx* muscles [48,49]. It is also known that a faulty metabolism impedes membrane repair and muscle regeneration [47,50]. This establishes/reinforces the proverbially downward spiral of degeneration and regeneration, and the ultimate failure of a regeneration characteristic of MD (Figure 1). Other mechanisms contributing to the spiral are reduced satellite cell function, fibrotic scar tissue, and chronic inflammation. By downward spiral, the positive feedback loops (indicated by the black circles in Figure 1) that these five mechanisms have upon each other is referred to.

In addition, the mitochondrial dysfunction can be subdivided into specific metabolic pathologies. The *mdx* muscle tissues have reduced ATP concentrations that are identified by a number of different techniques [51,52,53,54,55]. However, the causes of this decreased ATP are still not clear. One source could be the increased demand for ATP that is required for muscle regeneration [35]. In addition, the ATP production rate is drastically reduced in the *mdx* mitochondria that are isolated from the diaphragm and tibialis anterior muscles [56]. The authors also demonstrate that a portion of the decreased ATP phenotype occurs because of reduced Complex I activity. Open questions remain regarding how much of the DMD pathology is caused by the decreased ATP? And how much would replacing ATP and a fully functioning metabolism benefit MD patients? The research community must also identify how a sarcolemmal based mutation causes mitochondrial dysfunction. Recent data identifies that dystrophin and the dystrophin sarcoglycan complex is also found at the T-tubules [57,58,59], which is nearer to the mitochondria than previously thought. It may be that a large portion of the mitochondrial dysfunction is a result of the unbalanced Ca^++^ in the dystrophic muscle cell cytoplasm [56,60].

Among the metabolic changes occurring in dystrophic muscle is an upregulation of reactive oxygen species (ROS, [61]). This group of molecules have long been investigated for their toxicity to many macromolecules when they are inappropriately upregulated [39]. They are therefore considered to be pathogenic, and experiments—with variable efficacy—have been conducted to decrease the ROS in *mdx* and DMD, which has been discussed below. Monoamine oxidase [62], overactive NAD(P)H oxidases [63], and faulty electron transport chain complexes [64,65] are believed to contribute to the increased ROS levels (reviewed in [66]).

Muscular dystrophic animals and humans also have reduced and/or mislocalized neuronal nitric oxide synthase (nNOS, [67]). Usually, nNOS is held at the sarcolemma by its inclusion in the dystrophin glycoprotein complex, but it is absent from the sarcolemma in DMD and *mdx* [67]. These nNOS changes are pathogenic, although the data is unclear if it is the absence of nNOS at the sarcolemma or its relocalization to the cytoplasm that is pathogenic (reviewed in [68]). For example, nNOS is required for vessel dilation during exercise, which is absent in *mdx* mice [69] and DMD patients [70,71]. The mice and patients thus have localized ischemic events [61], further damaging the muscles. However, other data indicates that the correct localization of nNOS is not strictly required for mild pathology. There are some mildly effected Becker MD patients who lack nNOS at the sarcolemma [70]. To rule out increased NO within the cytoplasm as being pathogenic, double knockout *mdx* and nNOS mice were generated. These mice did not have a decreased pathology with respect to the *mdx* mice, indicating that additional NO within the cytoplasm is not pathogenic [72]. Multiple *mdx* nNOS overexpressing strategies have demonstrated a reduced *mdx* pathology, including reduced fibrosis, inflammation, and immune cell infiltration, as well as increased nitric oxide and force (reviewed in [68]). nNOS is included in this review because it is a metabolic molecule, which is increased with exercise, increased through activated AMP-activated protein kinase (pAMPK) signaling, and when in-excess causes dysfunction of the mitochondria, specifically within the electron transport chain [73]. NO is also central to the cellular entry of glucose [74] and a strong regulator of phosphofructokinase [75].

In normal adult skeletal muscle, Ca^++^ oscillates between 50 nM and 5 µM during excitation–contraction coupling. Many mechanisms are utilized to regulate the changes in Ca^++^ concentrations, the major ones are voltage-gated L-type calcium channels on the sarcolemma, and ryanodine receptors and SERCA on the sarcoplasmic reticulum. The first two of these channels cause the large Ca^++^ influx for a contraction, and then the SERCA reabsorbs the Ca^++^ into the sarcoplasmic reticulum. In addition, mitochondria function to buffer Ca^++^ extremes [76]. In dystrophic skeletal muscles, Ca^++^ levels are increased at both extremes and do not fluctuate as quickly [77]. Normally, Ca^++^ is a stimulator of oxidative phosphorylation to increase ATP during periods of increased contractions [78]. However, in DMD, there is clearly too much Ca^++^, beyond the normal oxidative phosphorylation stimulation range. Therefore, the feed-forward downward spiral of MD (Figure 1) is enhanced by these Ca^++^ surges. Damaged mitochondria cause a reduced Ca^++^ control, which further damages the mitochondria by activating the permeability transition pore [79], which causes even more Ca^++^ misregulation [80]. In support of this downward spiral are the progressive phenotypes of the disease and the progressive deterioration of the mitochondrial function with age in DMD patients [81] and *mdx* mice [82].

## 5. Possible Metabolic Therapeutic Avenues

The many experiments, both pre-clinical and clinical, that attempt to modify one or more of the above discussed metabolic pathways for improvement of the MD phenotype, will be discussed (Table 1). Because many of the targets result in changing multiple metabolic molecular pathologies, the pathways cannot be sorted to fit into the specific pathologies that are identified in Figure 1. Instead, these experiments are largely sorted by molecular target. The overlapping nature of many of these potential therapeutic targets will be discussed. It is important to note that nutritional and metabolic therapies target many of the pathogenic stages of MD (Figure 1). By inhibiting the disease progression at a number of stages, a more complete rescue of phenotype is possible.

## 6. Dietary Supplements 

Many researchers have investigated various dietary supplements to inhibit muscle pathology or strengthen non-diseased muscles, whether it be for athletes or against aging, type 2 diabetes, or MD [83,84,85,86,87,110,128,129,130,131,132,133]. In MD, increasing the percentage of slow fibers is often the goal, but not necessarily the only goal. Natural interventions such as diet are useful, with low if any side-effects interventions. One must be careful to remember that the metabolic aspects of the MD pathology are different than those of true metabolic diseases and aging. And although not all of these therapies have been tried in pre-clinical or clinical MD, the targets that the supplements are altering are speculated to also benefit the *mdx* mice and DMD patients. In addition, these supplements can be combined with other treatments, causing an additive benefit and/or allowing a decreased dose of the co-therapy pharmaceutical.

## 7. Polyphenol Supplement

It has been demonstrated that a low dose (0.5% by weight) of apple polyphenols increased the percentage of slow, Type 2 fibers in plantari muscles (from ~48% to ~58%, *p* = 0.02), after feeding it to rats for 8 weeks [83]. As expected, this change correlated with fatigue resistance in the treated rats. However, the intervention has not yet been tried in *mdx* or other dystrophic mouse models. From the overwhelming known benefits, it can be hypothesized that this intervention—resulting in increased slow Type 2 fibers—will benefit mice and humans with MD.

## 8. Amino acid Supplements

A number of investigators have analyzed l-arginine supplementation in both mice [110] and humans [84]. In the mice, the L-arginine administration caused an increase of NO, members of the dystrophin glycoprotein complex, and a functional increase of isolated muscles. In the humans, the L-arginine was administered in conjunction with metformin, and the patients’ muscles demonstrated increased mitochondria numbers, reduced oxidative stress, and four out of five patients improved their clinical scores, such as the two minute-walk-test and motor function measure.

Creatine supplementation starting at birth reduced the *mdx* pathology at 4 weeks old [85]. Furthermore, the benefits were demonstrated to include improvements to mitochondrial functions. This demonstrated at least a correlative link between improved mitochondrial function and reduced *mdx* phenotype.

Oral glutamine inhibited protein breakdown in DMD patients, but this did not lead to significant functional benefits [86,87].

Additional groups of scientists are investigating taurine supplementation. When given in drinking water to dams, well before pregnancy, the taurine levels were significantly elevated in the offspring. The dams and offspring were then maintained on the taurine supplements until euthanasia. Young, 4 weeks old, offspring showed significant tibialis anterior benefits in specific force and histology. However, older mice, 10 weeks old, did not demonstrate any benefits [128].

## 9. Fatty acid Supplements

Interesting publications indicate that including omega-3 supplements in diets significantly improved the MD phenotype. Marine fish are rich in omega-3, which includes eicosapentaenoic acid (EPA) and docosahexaenoic acid (DHA). These polyunsaturated fatty acids (PUFA) have been shown to reduce inflammation in other diseases and conditions. This strategy could therefore be highly beneficial against DMD and would also be highly useful as a co-therapy. Feeding MD hamsters flaxseed-derived omega-3 from birth until death significantly improved skeletal muscle histopathology [129]. Additional studies in *mdx* mice, both young [130] and old [131], benefitted from omega-3 treatment. The benefits include reduced ROS, increased grip strength, decreased fibrosis, and improved echo parameters. These benefits can be direct or as a result of decreased inflammation [132] and a shift from M1 to M2 macrophages [133]. These results were so promising that clinical trials were initiated. The initial reports from the trials have indicated a significant decrease in the markers of chronic inflammation in the 36 patients that had been receiving supplements for 6 months [134]. Future clinical trials will likely include identifying any functional—skeletal muscle strength, respiration, and cardiac—benefits.

AMP activated protein kinase (AMPK and the activated phosphorylated pAMPK) is a primary or secondary target for many of the metabolic MD therapies that are currently being considered (reviewed in [40,135]). In patients, pAMPK can be increased by a number of pharmacological or physiological approaches (reviewed in [44]). Most directly, pAMPK is increased physiologically through exercise and calorie restriction, and pharmacologically with Metformin or 5-aminoimidazole-4-carboxamide riboside (AICAR). There are many pAMPK-mediated MD benefits. pAMPK activates the signaling cascades, which increase slow fiber content [97,106]; increases utrophin, which compensates for dystrophin at the sarcolemma [136]; it is necessary for the exercise-activation and stretch-activation of nitric oxide synthase [137]; decreasing inflammation [122]; decreasing ROS [137]; and decreasing TGFβ signaling [138].

In addition, we demonstrated that the super-healing and MD-resistant mouse strain, named Murphy Roth’s Large (MRL), had significantly increased pAMPK levels in their skeletal muscle tissues [41,139]. These are wild type mice, not genetically manipulated or treated in any manner. In a not side-by-side comparison, the MRL mice appeared to be more resistant to MD than the mice that were treated with metformin [139,140] or AICAR [45,49,106]. Therefore, further work on the MRL mice is necessary in order to fully identify why these mice are so resistant to the fibrotic aspects of MD.

## 10. Exercise

Both anaerobic exercise (resistance training) and aerobic exercise (activities that increase breathing and heart rate, and often labeled as endurance exercise), affect the fiber type transition. Anaerobic exercise induces a higher percentage of fast fibers, up 28% in recreationally active men [141]. Alternatively, aerobic exercise elicits the slow fiber transition [142] that is beneficial in *mdx* mice [97] and DMD patients [143]. Aerobic exercise also increases slow muscle inducing signals, such as AMPK, SIRT1, and PGC-1α, which have protective benefits beyond fiber type switching [22]. SIRT1 has many beneficial functions. It is essential in the pathways that control metabolism, mitochondrial functions, autophagy, and limits inflammation [144]. Similarly, PGC-1α has many documented beneficial functions. PGC-1α is a master regulator of mitogenesis, and also promotes the adaptation of slow fiber characteristics [145].

Because exercise could potentially harm DMD patients, care must be taken in order to fine-tune the exercise intensity for maximum benefit, without detrimental side-effects. The reader is referred to the reviews of *mdx* and DMD exercise protocols in order to address some of these concerns [93,146].

Multiple studies have examined the phenotypic outcomes of *mdx* mice that have been subjected to exercise (reviewed in [89]). Low-intensity endurance exercise on a motorized Rota-Rod for 6 weeks, decreased the cell degeneration, inflammation, and necrosis in limb muscles [147]. Importantly, the diaphragms of the exercised *mdx* mice had increased regenerative areas and decreased necrosis assessed by histology, compared to sedentary *mdx* [89]. Voluntary wheel running also increased the mass and force of skeletal muscles [104,148,149]. In older mice (10 weeks old at start), a short bout of voluntary wheel running increased muscle damage [150]. These collected data are consistent with low intensity and voluntary exercise being beneficial for *mdx* mice, when started at a young age. Caution must be used when these data are to be applied in the clinics. The age and heart condition of the patient must be thoroughly evaluated before the initiation of a monitored exercise regime.

In addition to stimulating a beneficial fiber type transition, aerobic endurance exercise also stimulates satellite cells—the resident stem cells in skeletal muscles [151,152,153] and reviewed in [154]). Satellite cells are more numerous in slow muscles, such as soleus, than in naturally fast muscles, such as extensor digitorum longus [155]. Aerobic exercise increases AMPK, SIRT1, and PGC1-α, which are also known to activate satellite cells. Interestingly, when investigated by fiber type, Evans blue dye he endurance exercise-increased satellite cell numbers were only detected in Type I, slow fibers [151,156]. This may be another underlying characteristic of slow fibers that makes them somewhat resistant to MD pathology.

To the best of my knowledge, calorie restriction to increase pAMPK, mitochondria, and mitochondrial efficiency has not been attempted on *mdx* mice. And, even if effective, full patient compliance would be difficult to achieve. However, it may be worth trying the therapy in mice and then considering utilizing one of the type 2 diabetic therapies (such as Invokana, canagliflozin), which reduce the absorbed calories in patients. Thereby inducing calorie restriction in a manner that is better suited for effective patient compliance.

Metformin is an attractive treatment to increase pAMPK because it is FDA approved for type 2 diabetes and therefore safety clinical trials have already been conducted. In *mdx* mice Metformin increases PGC1α and utrophin [96]. A recent manuscript described a proof-of-principle Metformin with l-Arginine co-therapy regiment in a small cohort of five DMD patients [84]. The patients were less than 10 years old and were functionally and biochemically assessed before and after the 16 weeks of treatment. After the treatment, many of the phenotypic characteristics trended to improvement, including: (1) reduction of oxidative stress markers; (2) energy substrate usage shifted from carbohydrate to fatty acids; and (3) motor function measurements. However, because of the small cohort, only a few of these characteristics were statistically significant. Hopefully, a similar study will follow soon with an increased cohort size and perhaps a longer treatment period. The same group also investigated Metformin with l-Citrulline in a small cohort [157], the results are eagerly anticipated.

There are also many examples of scientists using AICAR to beneficially increase pAMPK in mouse models of MD. The mechanisms, by which AICAR increases pAMPK, are still under investigation, but clearly the inhibition of the electron transport chain is among the pathways. These AICAR-based pAMPK increasing investigations have greatly enhanced the knowledge of the breadth of pAMPK’s positive activities in *mdx* mice. For example, the stretch activation of NOS enzymes in cardiomyocytes requires dystrophin and pAMPK [137]. Four weeks of AICAR treatment causes a normalization of the calcium sensitivity of the MPTP in diaphragm muscle cells [45]. There was also a decreased histopathology and increased diaphragm force generating capacity. Interestingly, these benefits were not associated with an increased mitochondrial number or increased Utr expression, identifying additional pleiotropic benefits of pAMPK. The authors hypothesize that increasing pAMPK also increases autophagy and mitophagy to eliminate defective mitochondria [45].

AICAR is also currently in clinical trials for diabetes [158], obesity (clinicaltrials.gov identifier NCT02322073), and cancer (clinicaltrials.gov identifiers NCT01193959 and NCT01246778). If it is found safe and effective in increasing pAMPK in these trials, future AICAR trials will likely be conducted in DMD patients.

Because it increases PGC-1α, SIRT1, and AMPK, the natural phenol resveratrol has also been investigated for the treatment for a number of diseases, including MD (reviewed in [159,160]). In a side-by-side comparison, resveratrol improved exercised *mdx* muscle pathology (histopathology, oxidative stress, and creatine kinase) better than prednisolone did [161]. Because these therapeutics target different disease sites, co-therapies must be considered for finding the optimally beneficial treatment. Additionally, it was demonstrated that resveratrol improved the *mdx* muscle function when administered to young mice [162]. Resveratrol has now also entered clinical trials for multiple diseases and one hopes that DMD will soon be on the list of trials.

A relatively new method for increasing pAMPK has been identified in miR-499 [31]. Because this molecule is a microRNA (miR), it can play a directorial role in coordinating the mitochondrial metabolic and sarcomeric protein transitions that are required for fiber type changes. The transition is initiated by the expression of Myh7/Myh7b and the intronic miR-499. miR-499 then affects metabolic changes by increasing AMPK and PGC1α, therefore increasing the oxidative metabolism. Experiments involving knockout and transgenic mice were used to deduce this molecular cascade. Therefore, it appears that miR-499 coordinates the metabolic and sarcomeric aspects of skeletal muscle transition [31].

Deleting folliculin-interacting protein-1 (Fnip-1) pushes skeletal muscle fibers to slow fibers benefiting the *mdx* mice [100]. This is believed to occur through AMPK activation and the subsequent upregulation of PGC1α. However, as the mice lack B-cells and invariant natural killer T-cells [100]—known to decrease MD pathology—it is unknown which mechanism or if a combination of both mechanisms are responsible forthe decreased pathology.

A final method of increasing pAMPK in mouse models of MD occurred unexpectedly when the wild type MRL mouse line was bred to have γ-sarcoglycan null-mediated muscular dystrophy; the MRL mice were found to have an extremely mild pathology [42]. It was then shown that the MRL mice naturally contain increased pAMPK in their skeletal muscles [41]. As discussed above, increasing pAMPK has been demonstrated to decrease *mdx* pathology. Of course these mice also contain many other genetic differences that will enhance their mild phenotype. Therefore, these mice require further investigations.

A series of experiments from Dr. Jasmin’s laboratory indicate that increasing utrophin by a number of methods, protects *mdx* mice from pathology [40]. Increasing utrophin with transgenes demonstrated significant phenotype improvements [101,102]. In addition, a small molecule utrophin transcriptional enhancer—SMT C1100—significantly decreased the *mdx* pathology [102]. This molecule is now in a safety, tolerability, and pharmacokinetic clinical trial. The patients tolerated the pharmaceutical rather well, but—surprisingly—not as well as the healthy volunteers [163]. AICAR and metformin, which are known activators of pAMPK, also increased the utrophin expression. Interesting work in double knockout mice (*mdx* with utrophin null) identified a strict requirement of utrophin for pAMPK’s benefit [106]. However, others have shown that utrophin is not required for all of the benefits of pAMPK, because the diaphragm functional and histopathologic improvements were observed after the AICAR treatment without a corollary increase in utrophin levels [45]. The variability in the requirement for utrophin may be due to different muscle groups that were analyzed or the different manners of assessing requirements. Because of the large amount of positive data, SMT C1100 must be considered for co-therapies.

Altering NO levels and signaling are also being tested in *mdx* and DMD. NOS and the resulting nitric oxide (NO) are required for blood pressure homeostasis during exercise. Furthermore, in contrast to the wild type, *mdx* cardiomyocyte stretch does not activate NOS [137]. Increasing NO through l-arginine administration [110] or transgenically [75,107,108,109,164] recues portions of the functional deficit and pathology in *mdx* mice. A confounding data series comes from rescuing the *mdx* mice with a truncated dystrophin transgene, which restores nNOS to the sarcolemma, but—paradoxically—the mice have an increased histopathology [165]. The AICAR activation of AMPK causes the activation of NOS, independent of dystrophin and stretch, indicating a pharmaceutical method to bypass the missing dystrophin and nNOS [137]. 

In humans, some selective PDE5 inhibitors like Tadalafil and Sildenafil, and some non-selective PDE inhibitors like Pentoxifylline are being tested for their ability to beneficially increase NO. These function by decreasing the breakdown of cGMP, a downstream second-messenger of NO, and causing increased NO signaling, which should benefit the patients. Despite proving beneficial in *mdx* mice [166], the Sildenafil treatment in DMD and Becker MD patients did not provide any functional benefits [113,114]. Neither study assessed the increase of NO after Sildenafil, likely given the problems that were associated with quantifying NO. Also, Sildenafil cannot reproduce the normal, oscillating NO levels. Therefore, Sildenafil is not as beneficial as AICAR, likely because the benefits that are seen with AICAR go beyond increasing the NO levels.

Specifically increasing the amount of mitochondria also reduces the *mdx* pathology. This has been done by transiently increasing PGC1-α, which causes the normalization of multiple mitochondrial parameters including Ca^++^ buffering, mitochondrial permeability transition pore (MPTP) opening kinetics, and mitochondrial mass [48]. Although it is difficult to identify the amount of benefit that is provided by this particular mechanism, the mitochondrial volume is also increased by exercise [167].

Antioxidants are also being tested in mouse models and human patients. Significantly, two clinical trials with Allopurinol in DMD demonstrated benefits for the patients [118,119,120]. One of the studies lasted for 27 months, which demonstrated an improvement in 2 of the 10 treated patients versus 0 of the 7 untreated patients [120]. The authors cite variability in the patient population, especially their ages, as confounding the data. In addition, improvement is a very high bar for MD therapies; no further diminishment of function maybe a more appropriate, although it is an unsatisfactory end-point for clinical trials. An additional Allopurinol study lasted a total of 22 months. There was an initial benefit [119], which faded over time. The authors hypothesized this was due to a decrease in the effective dose for children over 9 years of age due to natural weight gain, after appropriate dose compensation, there was an additional period of effective treatment [118].

Adiponectin is the founding member of a novel class of signaling molecules termed adipokines, which are secreted from adipocyte—cells long considered devoid of signaling properties. Adiponectin reverses the diseased metabolic characteristics of type 2 diabetes and atherosclerosis, and very importantly has anti-inflammatory effects [168]. Although secreted from adipose tissue, the amount of circulating adiponectin does not correlate with a person’s percent fat mass. In *mdx* mice, the levels of adiponectin are significantly decreased [169]. When the adiponectin overexpressing mice were crossed with the *mdx* mice, inflammation and pathology diminished through an increase of the myogenic program. The authors also demonstrated that these beneficial effects were through the AMPK–SIRT1–PGC1α signaling cascade. Although changes in the metabolic characteristics were not assessed, increased AMPK–SIRT1–PGC1α signaling should cause an increased mitochondria number and a shift to slow fibers with the associated low pathology of those cells. The *mdx*/adiponectin transgenic mice had decreased levels of CD68, an M1 macrophage marker, and increased IL-10, which activates and is a marker for the anti-inflammatory M2 macrophages. The adiponectin animals also had decreased NF-κB. Interestingly, the authors also noted an increase in utrophin [169]. All of these characterizations strongly indicate that increasing adiponectin is a possible treatment of MD.

It has long been known that MPTPs are open inappropriately in *mdx* and DMD [123], likely due to the enhanced cytoplasmic Ca^++^ of the *mdx* and DMD muscle cells. Multiple methods to decrease this are known and have been investigated in the *mdx* and *mdx*/*utr* DKO mice. Cyclosporine A beneficially regulates the pore and is also an immunosuppressive, while Debio 025 just regulates the pore. Both of these compounds benefit *mdx* mice [84,124,125]. It has also been demonstrated that Debio 025 is more effective in reducing the *mdx* pathology and increasing function than prednisone, which is the current standard of care [170]. Other treatment options that have already been discussed, increasing pAMPK or increasing PGC-1α, also decrease the transition pore openness [45,48,111]. The effects could be direct or indirect; indirect because a reduction of pathology would cause the transition pore response to become more like normal.

## 11. Conclusions

There is great excitement in the muscular dystrophy (MD) field due to the large amount of preclinical and clinical trials that are currently underway. There are reasons to be optimistic regarding these future therapies. For one, many of the therapies have and continue to demonstrate great promise in preclinical experiments. Multiple co-therapies, which target multiple points of the MD progression pathway, have proven of benefit for the *mdx* mice. Two common metabolic targets are to increase AMPK and PPARβ/δ with AICAR and GW501516, respectively. The benefits of AICAR treatment were enhanced with the co-administration of GW501516 increased mitochondria, slow-fiber type, and Utr, and decreased membrane permeability, fibrosis, inflammation, and central nucleation [49,127]. Four weeks of the combined treatment regimen in two month old mice led to the expression of Type I genes, functional improvements, and the creatine kinase levels were reduced [127]. The other set of experiments initiated treatments in older, twelve week old mice [49]. These investigators also saw a decline in inflammation, percent central nuclei, and activated satellite cells, as well as an increase in limb strength. These experiments underscore the very important point that, in order to treat the multiple pathogenic pathways of MD, co-therapies must be considered. An additional co-therapy strategy using AICAR for 30 days, followed by a single acute exercise bout, was investigated [126]. The exercised *mdx* mice demonstrated increases in pAMPK, p38 phosphorylation, PPARδ, PGC-1α, and SIRT1. Paradoxically, the pre-treatment with AICAR blunted these favorable responses towards a slow-fiber transition; one would have hypothesized that there would be an additive effect of these two treatments. These data demonstrate the importance of pre-clinical testing of all therapies, including co-therapies.

Additional co-therapies can be envisioned to have benefit in preclinical and clinical trials. An example might be exon skipping to restore some dystrophin expression, perhaps coupled with a mitochondrial support molecule, such as Metformin, and an immune suppressor, such as low dose corticosteroids. These co-therapies would be beneficial by targeting multiple sites and would allow reductions of doses to avoid any side-effects (Table 2). Even if the therapies do not act synergistically, additive effects would greatly improve patients’ lives. In addition, because multiple sites are being targeted, one can envision many patient-specific combinations of co-therapies. Additional excitement comes from the knowledge that only 6–10% [171], 20% [172], or 40% [173] of dystrophin expression is required for significant pathology reduction. The differences in the amount of dystrophin restoration that is required for benefit is that different models and assessments were utilized.

Many of the nutritional and metabolic therapeutics that have been discussed for MD are already in clinical trials. Column four of Table 1 indicates those that are currently in clinical trials and the initial results of these trails. As the clinical trials move forward, there are also some difficulties confronting the MD research, and the clinical and patient community. One difficulty is that all muscles, including skeletal, diaphragm, and cardiac muscle cells, must be targeted. This causes the delivery of some of the most promising therapies to be problematic. In addition, as more and more clinical trials are recruiting patients, identifying naïve patients may also become problematic. This confirms the need for robust preclinical models and a standard that is set of phenotypic methods to verify that the patients are receiving the best evidence based medicine that is available.

## Figures and Tables

**Table 1:** Metabolic targets to treat muscular dystrophy.

Target	Rx	Mice	Humans	References
Dietary supplements	Apple polyphenols	Increases slow fibers		[83]
	Arginine & Metformin		16 weeks of co-treatment improved clinical scores in normal volunteers	[84]
	Creatine	Early treatment reduced *mdx* pathology		[85]
	Glutamine		10 treatment days did not alter protein degradation compared to amino acid controls	[86]
			4 months of treatment did not improve DMD pathology	[87]
Increase pAMPK	Exercise	Increases slow fiber types, PGC-1α, SIRT1	Increases slow fiber types, PGC-1α, SIRT1	Reviewed in [22]
		Increases utrophin in skeletal muscles after 12 weeks of voluntary wheel running		[88]
		Low intensity training improved *mdx* phenotype through inflammation inhibition in skeletal muscle and diaphragm.		[89,90]
			Exercise increased utrophin in skeletal muscles of normal people	[91]
			Submaximal exercise increases function without causing increased pathology	[92]
			DMD exercise reviews	[93,94]
			Assisted bicycle training maintains function without causing increased pathology	[95]
	Metformin	Increased PGC1α, utrophin.		[96]
	Metformin with l-arginine		16 weeks of treatment caused a trend to improved oxidative stress and function	[84]
	AICAR	Increased slow fiber types, PGC-1α, SIRT1		[97]
		Restored calcium-induced PTP opening to normal levels in diaphragm.		[45]
	Resveratrol	Modest pathology decline, increased Utr		[98,99]
	Transgenic overexpression of Mir-499	Reduced pathology		[31]
	Inhibit Fnip1	Reduced pathology		[100]
	Through breeding	Reduced fibrosis and functional decline		[41]
Increase utrophin	Transgenically	Significantly decreases pathology		[101,102]
	SMT C1100	Significantly decreases pathology		[103]
			Safe in healthy volunteers	[104]
			Tolerated in DMD patients, high degree of variability in serum SMT C1100 levels	[105]
	AICAR	Increases utrophin		[106]
	Metformin	Increases utrophin		[96]
Increase nNOS/NO	Transgenically	Decreased pathology		[68,107,108,109]
	l-Arginine	Reduced pathology		[110]
	PDE inhibitors, sildenafil	Reduced pathology in skeletal, including diaphragm and cardiac muscles. Increased slow fiber transition.	Acute treatment reduced exercise-associated ischemia.	[111] reviewed in [68,112]
			Two phase 3 trials have recently been completed but failed to demonstrate improvements, chronic treatment.	[113,114]
Increase PGC1α	Transgenic	Reduced pathology, increased slow fibers, mitochondria and Utr. Decreased CN, EBD and CK.		[34,48,115,116]
Support ATP generation ROS inhibitor	ASA		Improved ATP content and multiple functional parameters	[117]
	Allopurinol		Initial increased creatine phosphate and ATP, and most patients retained benefits after 22 months.	[118,119]
			Unclear benefit after 27 months of treatment.	[120]
Increase adiponectin	Transgenic overexpression of adiponectin	Increased slow fibers Increased utrophin Decreased pathology		[121]
	Treated DMD myotubes with adiponectin		Decreased inflammation and upregulated utrophin	[122]
Transition pore inhibitors	Cyclosporine A	Restored normal redox state in isolated *mdx* cell mitochondria		[123]
	PGC1α transgene	Normalized MPTP opening kinetics.		[48]
	AICAR	Normalized MPTP opening kinetics in the diaphragm.		[45]
	Sildenafil	Normalized MPTP opening kinetics in hearts.		[111]
	Debio 025	2 weeks of oral treatment, some structural and functional improvements in diaphragm and skeletal muscles.		[124]
	Genetically targeting cyclophilin D, or Debio 025	Reduces myofiber necrosis and pathology in Lama2 and delta-sarcolglycan deficient mice		[125]
Co-therapies	30 days AICAR followed by exercise	Paradoxically AICAR blunted the beneficial effects of exercise		[126]
	AMPK/PPARγ agonists	Histological and functional improvements		[49]
	Exercise with AMPK/PPARγ agonists	Functional improvement in the combination group		[127]

AICAR—5-aminoimidazole-4-carboxamide riboside; ASA—5-aminosalicylic acid; CK—creatine kinase; CN—central nuclei; DKO—double dystrophin and utrophin knockout mice; DMD—duchenne muscular dystrophy; EBD—Evans blue dye; Fnip-1—folliculin interacting protein 1; MD—muscular dystrophy; MPTP—mitochondrial permeability transition pore; nNOS—neuronal nitric oxide synthase; NO—nitric oxide; pAMPK—phosphorylated AMP activated protein kinase; PDE—phosphodiesterase; PGC1-α—peroxisome proliferator-activated receptor γ coactivator 1-α; SIRT1—NAD-dependent deacetylase sirtuin-1; TGFβ—transforming growth factor beta 1; utr—utrophin.

**Table 2:** Multiple muscular dystrophy targets.

Targeted Molecular Pathway	Therapy
Improve sarcolemmal strength, dystrophin expression	Omega-3 FTY720 Utrophin upregulation Exon skipping Cell based
Decrease inflammation	Corticosteroids NF-κb inhibition TGFβ inhibition Antioxidants
Improve mitochondrial function	AICAR Metformin Exercise Antioxidants
Decrease fibrosis	Losartan TGFβ inhibition
Improve satellite cell functions	Cell based

This table uses the five main pathogenic steps of MD disease progression, as introduced in Figure 1. Effectively targeting multiple steps of the disease progression pathway would provide the most patient benefit. As stated before, many of the treatments target multiple molecular pathways so the therapies were put into the current prominent target.

## Abbreviations

AICAR	5-aminoimidazole-4-carboxamide riboside
ASA	5-aminosalicylic acid
CK	creatine kinase
CN	central nuclei
DKO	double dystrophin and utrophin knockout mice
DMD	duchenne muscular dystrophy
EBD	Evans blue dye
Fnip-1	Folliculin interacting protein 1
MD	muscular dystrophy
MPTP	Mitochondrial permeability transition pore
nNOS	neuronal nitric oxide synthase
NO	nitric oxide
pAMPK	phosphorylated AMP activated protein kinase
PDE	phosphodiesterase
PGC1-α	Peroxisome proliferator-activated receptor γ coactivator 1-α
SERCA	sarco/endoplasmic reticulum Ca^2+^-ATPase
SIRT1	NAD-dependent deacetylase sirtuin-1
TGFβ	transforming growth factor beta 1
Utr	utrophin
